# Dataset on the chemical composition and bioactive compound of estuarine seaweed *Gracilaria* from four different cultivation area in Java and Lombok Island, Indonesia

**DOI:** 10.1016/j.dib.2024.110825

**Published:** 2024-08-19

**Authors:** Sri Purwaningsih, Wahyu Ramadhan, Weni Trinova Nabila, Eka Deskawati, Hamzah Moch Baabud

**Affiliations:** a Department of Aquatic Product Technology, Faculty of Fisheries and Marine Sciences, IPB University. Jl Agatis Kampus IPB Dramaga, Bogor, West Java 16680, Indonesia; b Center for Coastal and Marine Resources Studies (PKSPL), International Research Institute for Maritime, Ocean, and Fisheries (i-MAR), IPB University, Bogor, West Java 16127, Indonesia; c PT. Kappa Carrageenan Nusantara, Jl. Raya Pasuruan-Malang Km.10, Kejayan, Pasuruan, East Java 67172, Indonesia

**Keywords:** Bioactive compounds, Estuarine macroalgae, Proximate composition, Pigments, Seaweed

## Abstract

The data examines the evaluation of the quality of *Gracilaria* seaweed as the main raw material of various industrial products. *Gracilaria*, a red seaweed, serves as the primary ingredient in the agar industry and is subsequently utilized in food, biotechnological fields, nutraceuticals, and pharmaceutical applications due to its rich nutritional and bioactive compounds beneficial for human health. In fact, this seaweed has been cultivated in many regions and countries, especially in Indonesia. Several areas, particularly Java and Lombok Island, are known as the primary producers of *Gracilaria* seaweed and its derivatives in Indonesia. However, the current state of research lacks comprehensive exploration regarding the relationship or correlation between cultivation areas and the resultant quality of derived seaweed products. It is especially valuable to investigate the dataset concerning its nutrition and bioactive profile. Thus, this study aims to investigate and provide the chemical composition and bioactive compound of Estuarine Seaweed *Gracilaria* from four different cultivation areas in Java and Lombok Island in Indonesia. There are three areas in Java, specifically Karawang, Situbondo, and Pasuruan, and one area in Lombok, as the main location of sampling. These seaweed samples were then evaluated for their proximate composition, dietary fiber, selenium, iodine, carotene, antioxidant, and bioactive compound profiles. *Gracilaria* seaweed from Lombok Island, Situbondo, Pasuruan, and Karawang displayed moisture content in the range of 9-11%, ash content of 5-6%, fat content ranging from 0.26-0.62%, protein content between 9-17%, and carbohydrate content varying from 64-73%. The content of seaweed from Karawang, Pasuruan, Situbondo, and Lombok were recorded as 66.35%, 59.94%, 57.41%, and 72.56%, respectively. The analysis revealed that *Gracilaria* from the Lombok area had a selenium content of 18.82 mcg/100 g, whereas Karawang Seaweed showed 31.04 mcg/100 g of selenium. The Situbondo area exhibited iodine content (19676.96 mcg/100 g), while the Lombok area had iodine content (10588.19 mcg/100 g). Additionally, the carotene pigment content in *Gracilaria* ranged from 11.64 to 16.95 mg/kg. *Gracilaria* seaweed from the Lombok area displayed an IC50 value of 17.96 ppm for antioxidant activity and 26.82 ppm for alpha-glucosidase inhibitor activity. In contrast, *Gracilaria* samples from the Karawang area exhibited IC_50_ values of 25.44 ppm for antioxidant activity and 33.37 ppm for alpha-glucosidase inhibitor activity. A bioactive compound was also detected in *Gracilaria*, recognized as phlorotannin. The significance of these data extends to the selection of seaweed sources and conditions for potential applications, benefiting both the seaweed farming and research communities. Eventually, these findings data can be utilized for further testing and evaluation of seaweed as a raw material for nutraceutical supplements, functional foods, and sustainable biomaterials.

Specifications Table

**Table:** 

Subject	Food Science; Aquatic Science
Specific subject area	Fisheries Science, Macroalgae bioactive and proximate composition.
Type of data	Raw, Table, Graph
Data collection	- Seaweed samples were collected from four locations representing the four major Sargassum seaweed culture location points in Indonesia. - Seaweed samples were then harvested at the fixed time of harvesting, with details listed within the method. - Samples were washed and separated for purity. They were then transferred to the IPB University Campus in Dramaga, Indonesia for storage in dried conditions in the laboratory. - In the laboratory, each sample was extracted and analyzed to obtain the chemical and bioactive profiles from these samples.
	Seaweed was extracted and its chemical and bioactive profiles were investigated using several instruments; distillation unit (Buchi K-355), dryer (Getra FD-30), blender (Philips HR-2115), Inductively Coupled Plasma – Mass Spectrometer (Thermo Scientific™ iCAP™ RQ ICP-MS), UV-VIS Spectrophotometer (Shimadzu UV-1900i Series), Centrifugation (HIMAC-CR, no. R12A6904357D0, Hitachi, Japan), Microplates reader (Thermo Fisher Scientific, Waltham, MA, USA), hot plate stirrer (Corning PC-420 D), Gas Chromatography (Shimadzu C118047, Kyoto, Japan), analytical scale (SF-400 C), and oven (DHG-9053A, Jiangsu, China).
Data source location	There are four districts in Indonesia, where *Gracillaria* seaweed samples were collected: Kawarang, West Java (5°56′52.8"S 107°05′54.0"E) Pasuruan, East Java (7°35′48.9"S 112°51′59.1"E) Situbondo, East Java (7°37′52.9"S 114°00′36.8"E) Lombok, West Nusa Tenggara (8°46′22.9"S 116°03′29.4"E
Data accessibility	Repository name: Mendeley Data Data identification number: 10.17632/yg3cm325py.2 Direct URL to data: https://data.mendeley.com/datasets/yg3cm325py/2

## 1 Value of the Data

• The data provides the chemical and bioactive profile from four different districts in the major cultural center of Gracilaria seaweed in Indonesia. The database on the chemical and bioactive comparison from different areas in Indonesia is limited, and it will mostly be beneficial for the seaweed industry.
• Researchers working on seaweed postharvest may benefit from this dataset as a benchmark in the evaluation of the best area or district for Gracilaria culture. Researchers can use the chemical compounds of seaweed and bioactive properties in this dataset to understand the quality and further application of Gracilaria, for agar-based food or chemical and pharmaceutical research and development.
• The significance of these findings extends to the selection of seaweed sources and conditions for potential use, either as further intermediate material for nutraceutical supplements or as agar material for functional foods.
• This dataset can be beneficial for researchers who will use it for further applications of seaweed as specific bioactive compounds and for conducting in vitro and in vivo research steps. Researchers in marine biology and environmental science can use this dataset to conduct comparative studies on regional biodiversity and environmental impact assessments. Meanwhile, those in food science and bioengineering could explore new applications for Gracilaria's bioactive compounds in functional foods and sustainable materials.
• Researchers, program managers, and policymakers in government working on tropical seaweed may benefit from this dataset to improve fisheries and management activities, especially for integrating this culture with a multicultural system. By integrating this data, information on seaweed can also evaluate Indonesia's seaweed consistency and sources to assure this material as a global product in the market. Researchers can directly apply this dataset in the development of new Gracilaria-based products, optimizing cultivation techniques for enhanced yield and bioactivity, thus bridging the gap between fundamental research and practical applications.
• Development of Indonesian database on seaweed properties will be supported by the dataset, especially the chemical and bioactive compound of these harvested Gracilaria seaweeds. By providing a shared resource, this dataset can encourage collaborative research efforts among scientists from various institutions and countries, fostering a more integrated and comprehensive understanding of Gracilaria seaweed.

## 2 Background

Seaweed, particularly Gracilaria, holds significant importance across various industries due to its multifaceted properties and rich nutritional content [1,6]. This versatile marine resource finds application not only in food but also in pharmaceuticals, biotechnology, nutraceuticals, and other industrial sectors. Gracilaria, as an estuarine seaweed species, is renowned for its abundance of bioactive compounds and nutrients beneficial for human health. Its high nutritional value, coupled with functional properties like gelling and thickening, makes it a valuable ingredient in diverse products. From food additives to cosmetics and pharmaceuticals, Gracilaria contributes to an array of formulations. Furthermore, the cultivation and utilization of Gracilaria support livelihoods in coastal communities, particularly in regions like Indonesia where seaweed farming is prevalent. Understanding the status, culture area and function of Gracilaria seaweed, is crucial for sustainable resource management and economic development in these areas. In this data article, we examine the quality characteristics of the Gracilaria seaweed, by analyzing the parameters of proximate composition, iodine, selenium, phlorotannin and carotene value from four districts in Indonesia. Additionally, we investigate the antioxidant, inhibitor alpha-glucosidase activity and bioactive profile in *Gracilaria* seaweed. Eventually, this data article anticipates valuable insights for various industries seeking to harness the seaweed potential.

## 3 Data Description

**Fig. 1:**
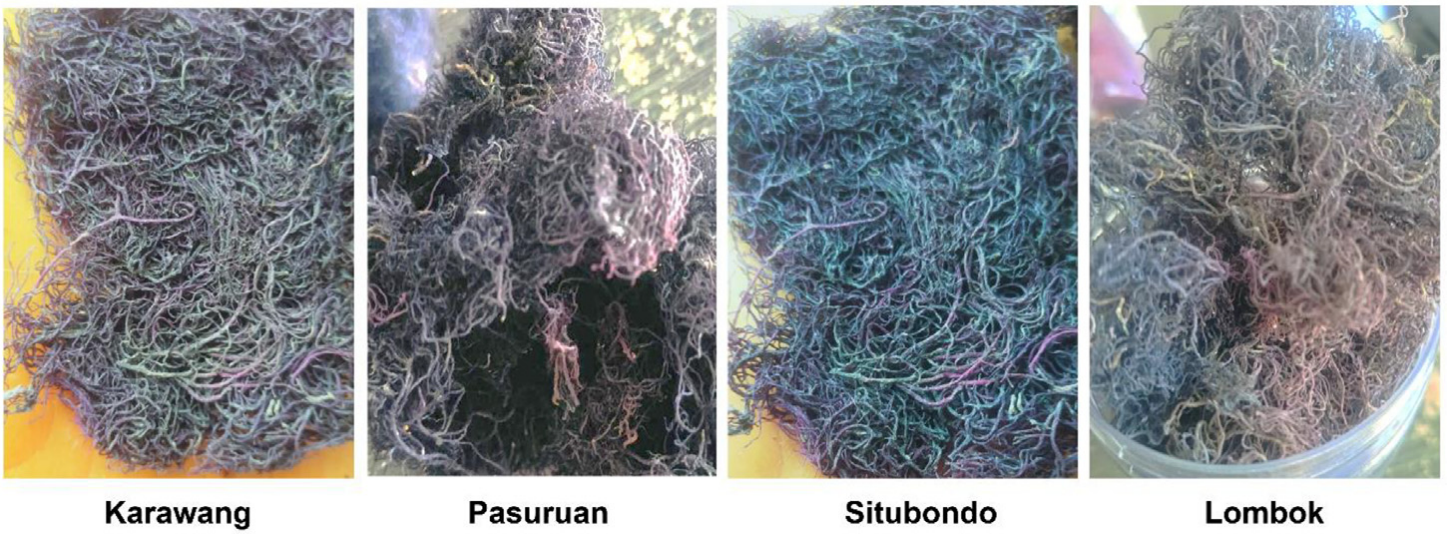
Photograph of dried seaweed samples collected from four different sampling areas.

**Fig. 2:**
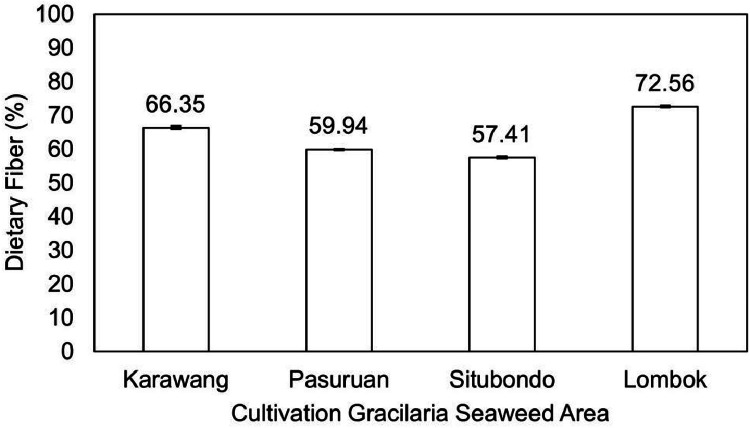
Dietary fiber content of Gracilaria seaweed from Java (Karawang, Pasuruan, Situbondo) and Lombok Island, Indonesia.

**Fig. 3:**
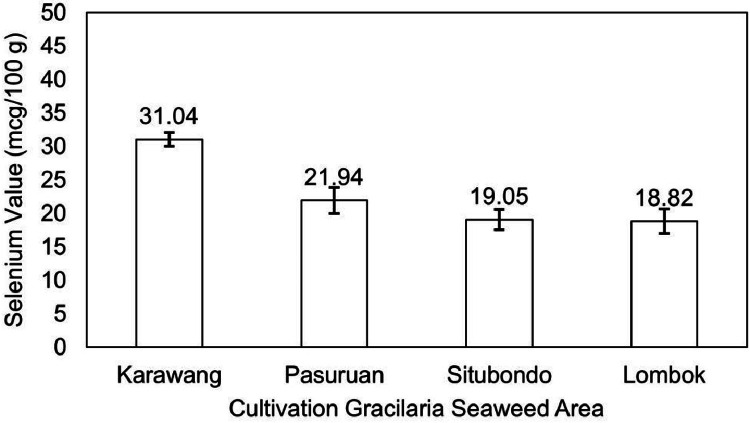
Selenium value of Gracilaria seaweed from Java (Karawang, Pasuruan, Situbondo) and Lombok Island, Indonesia.

The present study examined the proximate components (Table 1) and dietary fiber in Gracilaria seaweed from four different areas (Fig. 1). Table 1 provides a comprehensive overview of the water, ash, fat, protein, and carbohydrate content of each seaweed sample. Notably, Gracilaria seaweed from Lombok Island exhibited the highest ash (6.58%), fat (0.62%), and dietary fiber (72.56%) content (Fig. 2), while having a lower water content compared to the other samples. The water content of Gracilaria seaweed from Java (Karawang, Situbondo, and Pasuruan) ranged from 11.34% to 11.83%, whereas the sample from Lombok contained 9.51% water. Furthermore, the highest protein content was observed in Gracilaria from the Pasuruan area (17.71%), followed closely by Gracilaria from the Situbondo area (17.54%). In addition to the proximate components, the study also investigated selenium value of Gracilaria as presented in Fig. 3. The analysis revealed that Gracilaria from the Lombok area had the lowest selenium content at 18.82 mcg/100 g.

**Table 1.** Proximate composition (moisture, ash, fat, protein, and carbohydrate content) of Gracilaria seaweed.

Cultivation area	Proximate Composition				
	Moisture (%)	Ash (%)	Fat (%)	Protein (%)	Carbohydrate (%)
Karawang	11.34±0.18	5.54±0.09	0.26±0.01	9.43±0.23	73.43±0.14
Pasuruan	11.47±0.20	5.17±0.06	0.34±0.01	17.71±0.40	65.31±0.27
Situbondo	11.83±0.21	5.76±0.18	0.32±0.01	17.54±0.42	64.54±0.80
Lombok	9.51±0.22	6.58±0.15	0.62±0.01	10.73±0.23	69.54±0.05

**Fig. 4:**
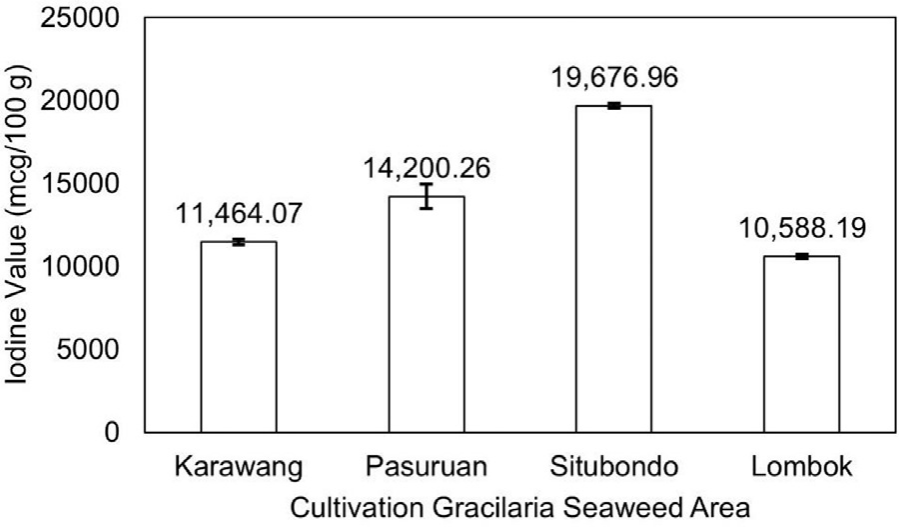
Iodine value of Gracilaria seaweed from Java (Karawang, Pasuruan, Situbondo) and Lombok Island, Indonesia.

**Fig. 5:**
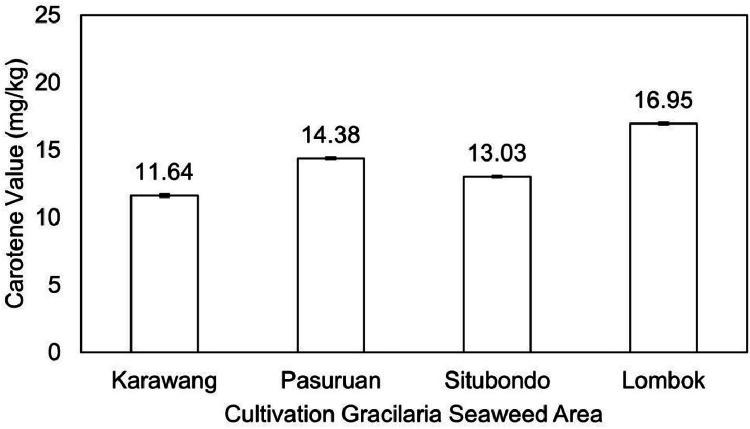
Carotene value of Gracilaria seaweed from Java (Karawang, Pasuruan, Situbondo) and Lombok Island, Indonesia.

**Fig. 6:**
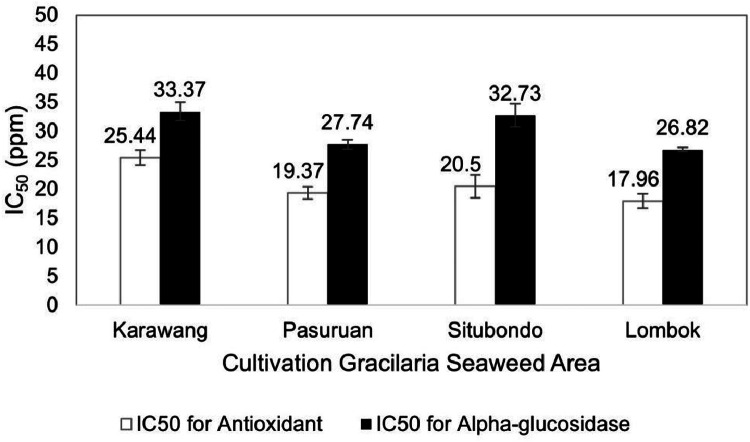
IC_50_ value of Gracilaria seaweed from Java (Karawang, Pasuruan, Situbondo) and Lombok Island, Indonesia for antioxidant (□) and alpha glucosidase (■).

Fig. 4 illustrates the iodine content of Gracilaria seaweed from the four cultivation areas. The Lombok area exhibited the lowest iodine content (10588.19 mcg/100 g), while the Situbondo area had the highest content (19676.96 mcg/100 g). Additionally, the carotene pigment content was highest in Gracilaria from the Lombok area (16.95 mg/kg) and lowest in Gracilaria from the Karawang area (11.64 mg/kg), as depicted in Fig. 5. Furthermore, Fig. 6 presents the antioxidant and alpha-glucosidase inhibitor activity of Gracilaria seaweed, as determined by the IC_50_ value. The IC_50_ value represents the concentration required for 50% inhibition, indicating the antioxidant and alpha-glucosidase inhibitor activities. The Gracilaria seaweed from the Lombok area displayed the lowest IC_50_ value compared to the other samples, with values of 17.96 ppm for antioxidant activity and 26.82 ppm for alpha-glucosidase inhibitor activity. Notably, all four samples exhibited IC_50_ values below 50 ppm, with the highest values recorded as 25.44 ppm for antioxidant activity and 33.37 ppm for alpha-glucosidase inhibitor activity in the Karawang area.

Phlorotannin was also detected in these areas, with data analysis showing the % area at specific retention times of 84.58, 83.23, 84.45, and 80.69% for Karawang, Pasuruan, Situbondo, and Lombok, respectively [2].

## 4 Experimental Design, Materials and Methods

### 4.1 Site description

The site on the First Island is situated in both the western and eastern parts of Java Island, encompassing Karawang in the west and Situbondo and Pasuruan in the east. These sites are predominantly estuarine areas where the primary pond culture of Gracilaria is centralized. Lombok Island was then selected as the second island with a central seaweed industry. Sekotong became the district area in Central Lombok, where estuarine conditions were identified. All samples were obtained in July-August 2023.

### 4.2 Seaweed sampling and sample preparation

In each location, we collected 3 samples within a week during the dry season of July-August 2023. Seaweed samples were randomly obtained within 100×100 cm transect plots. In each plot, we took seaweed samples from five points using transect sampling tools. Gracilaria seaweed in all areas is commercially cultivated in pond areas by seaweed farmers, employing a multi-culture system that includes seaweed, milkfish, and shrimp simultaneously within the same pond area. The water has a salinity level of approximately 19%. Seaweed is first harvested at the age of 3 months, with subsequent harvests conducted every 45 to 50 days after the previous harvest. The initial harvest at each location is fixed for the first harvest.

The harvesting process involves collecting a portion of the existing seaweed to be dried under sunlight for around 2 days if there is sufficient sunlight, or 3 days if sunlight is insufficient, or until the moisture content reaches about 16%. The seaweed is then ready to be transported to the collection warehouse and then transferred to the laboratory. Harvested seaweed from each area shares fixed conditions, including a minimum harvest age of 45 days, ranging in color from dark green to black, cylindrical talus without flattening, free from moss, with a maximum moisture content of 16%, and a maximum filth content of 5%. The number of ponds and samples was 3 ponds and 3 samples at each location.

Upon arriving at the laboratory, samples of Gracilaria seaweed are cleaned again with water to eliminate any remaining traces of sand, epiphytes, and other surface pollutants. The samples are arranged in a single layer on trays and then dried again in the dryer (Getra FD-30), set to maintain a temperature of 60°C for six hours. The dried samples are finely ground using a blender (Philips HR-2115) to ensure the production of homogeneous seaweed powder. The seaweed powder is packed into airtight plastic bags and kept at ambient temperature before analysis.

### 4.3 Determination of proximate composition

Proximate composition was determined according to the AOAC methods 930.05, 930.04, 2001.11.2005, for protein (total nitrogen × 6.25), ash, and moisture content, respectively [3]. Protein was determined by the Kjeldahl method, using a distillation unit (Buchi K-355). Lipid content was determined according to the AOAC methods 923.05, respectively by gravimetric method, using Soxhlet apparatus. Carbohydrate content was determined with a by-difference method.

### 4.4 Total dietary fiber

Total dietary fiber was determined according to the AOAC 985.29-1986 (2003) [4]. Dietary fiber content was determined by enzymatic-gravimetric method, using α-amylase and amyloglucosidase enzyme.

### 4.5 Selenium and iodine value

Selenium and iodine were analyzed by using Inductively Coupled Plasma – Mass Spectrometer (Thermo Scientific™ iCAP™ RQ ICP-MS).

### 4.6 Carotene content

Carotene was determined according to the AOAC 970.64-1974 by spectrophotometric method, respectively [5]. The prepared samples were then analyzed by using UV-VIS Spectrophotometer (Shimadzu UV-1900i Series) with wavelength 436 nm.

### 4.7 Antioxidant activity

The antioxidant content testing begins with the preparation of an extract solution. Seaweed extract with the addition of seaweed, totaling 10 mg, is dissolved in 10 mg of PA methanol to obtain concentrations of 75, 100, 150, 200, and 250 ppm. The stock solution at each concentration is further diluted with methanol solvent to a total volume of 10 mL. The next step involves the preparation of a DPPH solution with a 1:3 ratio. From each concentration stock solution, 1.5 mL is taken and added to test tubes containing 0.5 mL of DPPH solution. The solution mixtures are then incubated at room temperature in the dark for 30 minutes. After incubation, measurements are taken using a UV-Vis spectrophotometer at a wavelength of 517 nm. The percentage inhibition is determined using the formula:

(1) Inhibition(%)=(Absorbanceblank−Absorbancesample)/(Absorbanceblank)×100%.

The results are input into a regression equation with the sample concentration as the abscissa (X-axis) and the percentage inhibition value of the antioxidant as the ordinate (Y-axis). The linear regression equation obtained in the form of y = a(x) + b is used to find the Inhibition Concentration 50% (IC50) of each sample by expressing the value of y as 50 and the value of x obtained as the IC50.

### 4.8 Inhibitor alpha glucosidase

1 mg of α-glucosidase enzyme was dissolved in 100 mL of 100 mM phosphate buffer (pH 7.0). Subsequently, 200 mg of bovine serum albumin, dissolved in 100 mM phosphate buffer (pH 7.0), was added. Prior to use, 1 mL of the enzyme solution was diluted 25 times with phosphate buffer (pH 7.0). The reaction mixture comprised 250 µL of 20 mM p-nitrophenyl α-D-glucopyranoside as the substrate, 490 µL of 100 mM phosphate buffer (pH 7.0), and 10 µL of sample solution with varying concentrations of 50, 100, 200, 400, and 800 ppm in 10 µL dimethyl sulfoxide (DMSO). The reaction mixture was incubated in a water bath at 37°C for 5 minutes, followed by the addition of 250 µL of enzyme solution, and further incubation at 37°C for 15 minutes. The enzyme reaction was terminated by adding 1000 µL of 200 mM sodium carbonate. The reaction products were then read for absorbance at a wavelength of 400 nm. Acarbose tablets (Glucobay), dissolved in a 1:1 mixture of buffer and 2 N HCl, with a concentration of 1% w/v, were used as the positive control. The precipitate was collected by centrifugation, and 20 µL of the supernatant was added to the reaction mixture as in the sample. The reaction results were measured using a UV spectrophotometer at a wavelength of 400 nm. Sample and positive control tests were performed in duplicate for comparison with the sample under investigation.

(2) PercentageInhibition=K−(S1−S0)/K×100

Where:

• *K* = absorbance of the negative control
• *S1* = absorbance of the sample with enzyme addition
• *SO* = absorbance of the sample without enzyme addition

### 4.9 Bioactive compound

The extraction of *Gracilaria* sp. referred to Purwaningsih et al. [6] involves a single maceration method with ethanol solvent, using a ratio of 1:6 *Gracilaria* sp. to extract for 48 hours. The maceration result is filtered through Whatman No. 42 paper and concentrated using a rotary evaporator at 40°C until a paste is formed. The paste is then prepared for the evaluation of active components, particularly phlorotannin, using GC-MS.

## Limitations

All samples were harvested completely during the dry season across all locations. Therefore, variations and different phenomena observed during the rainy season could lead to different results. The current data does not present the temporal effects of seasonal changes on seaweed quality.

## Ethics Statement

This work does not involve human subjects and animal experiments.

## Credit Author Statement

**Sri Purwaningsih**: Funding acquisition, Conceptualization, Methodology, Supervision. **Wahyu Ramadhan**: Methodology, Writing – review & editing, data preparation. **Weni Trinova Nabila**: Writing – review & editing, field sampling, data preparation. **Eka Deskawati**: field sampling, Supervision. **Hamzah Moch Baabud**: field sampling, Supervision.

## Declaration of Competing Interest

The authors declare that they have no known competing financial interests or personal relationships that could have appeared to influence the work reported in this paper.

## Data Availability

Chemical and Bioactive Compounds of Estuarine Gracilaria Seaweed from Indonesia (Original data) (Mendeley Data).
